# A novel N95 respirator with chitosan nanoparticles: mechanical, antiviral, microbiological and cytotoxicity evaluations

**DOI:** 10.1186/s11671-023-03892-8

**Published:** 2023-09-21

**Authors:** Marcela Guimarães Landim, Marcella Lemos Brettas Carneiro, Graziella Anselmo Joanitti, Carla Tatiana Mota Anflor, David Dobkowski Marinho, José Filipe Bacalhau Rodrigues, Wladymyr Jefferson Bacalhau de Sousa, Daniel de Oliveira Fernandes, Beatriz Ferreira Souza, Alicia Simalie Ombredane, Jessica Catarine Frutuoso do Nascimento, Gisela de Jesus Felice, Aline Midori Adati Kubota, Juliana Simas Coutinho Barbosa, John Hideki Ohno, Solomon Kweku Sagoe Amoah, Lindomar José Pena, Glécia Virgolino da Silva Luz, Laise Rodrigues de Andrade, Willie Oliveira Pinheiro, Bergmann Morais Ribeiro, Fábio Rocha Formiga, Marcus Vinícius Lia Fook, Mário Fabrício Fleury Rosa, Henry Maia Peixoto, Rodrigo Luiz Carregaro, Suélia de Siqueira Rodrigues Fleury Rosa

**Affiliations:** 1 University of Brasília (UnB), Brasília, 70910-900 Brazil; 2 Northeast Laboratory for Evaluation and Development of Biomaterials (CERTBIO), University of Campina Grande, Campina Grande, Paraiba Brazil; 3 Aggeu Magalhaes Institute (FIOCRUZ), Recife, Pernambuco Brazil; 4 MCI Ultrasonica LTDA, Av. Campinas, 367 - Arraial Paulista, Taboão da Serra, São Paulo Brazil; 5 National Laboratory for Scientific Computing, Petrópolis, RJ Brazil

**Keywords:** Coronavirus, Antiviral, Facemask, Chitosan, Nanomaterial, Biopolymer

## Abstract

**Background:**

It is known that some sectors of hospitals have high bacteria and virus loads that can remain as aerosols in the air and represent a significant health threat for patients and mainly professionals that work in the place daily. Therefore, the need for a respirator able to improve the filtration barrier of N95 masks and even inactivating airborne virus and bacteria becomes apparent. Such a fact motivated the creation of a new N95 respirator which employs chitosan nanoparticles on its intermediate layer (SN95 + CNP).

**Results:**

The average chitosan nanoparticle size obtained was 165.20 ± 35.00 nm, with a polydispersity index of 0.36 ± 0.03 and a zeta potential of 47.50 ± 1.70 mV. Mechanical tests demonstrate that the SN95 + CNP respirator is more resistant and meets the safety requisites of aerosol penetration, resistance to breath and flammability, presenting higher potential to filtrate microbial and viral particles when compared to conventional SN95 respirators. Furthermore, biological in vitro tests on bacteria, fungi and mammalian cell lines (HaCat, Vero E6 and CCL-81) corroborate the hypothesis that our SN95 + CNP respirator presents strong antimicrobial activity and is safe for human use. There was a reduction of 96.83% of the alphacoronavirus virus and 99% of H1N1 virus and MHV-3 betacoronavirus after 120 min of contact compared to the conventional respirator (SN95), demonstrating that SN95 + CNP have a relevant potential as personal protection equipment.

**Conclusions:**

Due to chitosan nanotechnology, our novel N95 respirator presents improved mechanical, antimicrobial and antiviral characteristics.

## Background

By September 2022, Brazil ranked second in total number of deaths and third in cumulative total cases worldwide due to the spread and action of the new coronavirus (SARS-CoV-2) pandemic and its variants [1–3]. The Brazilian Unified Health System (SUS) is a major player in the intervention scenario to control the pandemic, and responsible for approximately 75% of the healthcare attention given to the Brazilian population. Notwithstanding, SUS has faced challenges such as the scarcity of personal protective equipment (PPE), medicines, oxygen supplies, diagnostic testing for SARS-CoV-2, as well as the saturation of hospitals and collapse of the health system [1, 4, 5].

During the pandemic, several SARS-CoV-2 lineages [Alpha (B.1.1.7), Beta (B.1.351), Gamma (P.1), Delta (B.1.617.2) and Omicron (B.1.1.529)] presented a high rate of circulation. Consequently, the use of adequate PPEs is deemed to minimize viral circulation and transmission [1, 6]. According to Rosa et al. [7], countries had to organize their flow of production and innovation to ensure adequate attention to the population's health demands. Domestic responses to reduce the pandemic’s impact encourage the development of solutions that do not depend on importing health products and supplies. Since the production and distribution of vaccines was limited [6] and the need for vaccine booster shots and even annual revaccination has been considered, it is essential to provide other preventive strategies such as social distancing, patient isolation, facial masks and hand hygiene to highly exposed populations, such as healthcare workers. Hence, considering the Brazilian scenario, the Brazilian Health Regulatory Agency (ANVISA) adopted the N95 semi-facial respirator as the recommended PPE, requiring at least a facepiece with type 2 filter (PFF2) and with 95% filtration capacity for solid, liquid or oily particles and filtration efficiency of 98.5% for bacteria (0.2–1.5 µm) [8].

It is worth noting that N95 respirators with FFR filter (“filtering facepiece respirator”) or similar, have reduced capacity to filter particles approximately 0.1–0.3 µm in size [9–12] The study of Flaxman et al. [13] investigated how implementations of non-pharmaceutical interventions affected the transmission of SARS-CoV-2 using a model that estimates transmission from observed deaths. The results show that the primary non-pharmaceutical interventions and lockdowns have significantly reduced transmission. Their analysis found that only the lockdowns are identifiable and that it has a substantial effect (81%, CI 95% 75–87%) of reduction in the rate of transmission [13]. Continued non-pharmacological interventions such as wearing masks or respirators should be considered for keeping the transmission of SARS-CoV-2 under control.

The type A Influenza virus, currently known as H1N1 or Swine Flu due to mutations that occurred in its transmission phases, was initially identified in Mexico in February 2009 and soon spread on a global scale [14]. In the period of 4 months, the transmission followed high rates spreading to more than 120 countries, thus causing the first pandemic of the twenty-first century.

According to a study published in December 2020 [15], the basic reproduction number (*R*_0_) of COVID-19 has been approximately 3 (95% CI 2.65–3.39), which is superior to that of influenza A (*R*_0_ = 1.3–1.7). Through the data collected by the notifiable diseases information system (SINAN) [16], between 2013 and 2016 in Brazil, there was a considerable increase in the transmission of Influenza A, considering the year 2016 that with the highest incidence and post-pandemic mortality cases.

Nevertheless, the SARS-CoV-2 and H1N1 viruses are spherical in shape, with viral particle diameter ranging between 50 and 200 nm [17, 18]. A PPE associated with nanofilm was considered more effective when compared to traditional ones due to the need of filtering out aerosol particles in the range of 10 nm to 10 μm, where particles relevant for transmission of respiratory viruses lies [19, 20]. Previous studies have demonstrated the applicability of nanotechnology in masks to tackle infectious agents [10], using materials such as graphene oxide, silver oxide and chitosan nanoparticles [21–26].

Several studies reported the use of chitosan as a low-cost natural cationic polymer derived from chitin, which is biodegradable, biocompatible, non-toxic and displaying antimicrobial activity [27, 28]. Chitosan has been associated with virucidal activity and inactivation of viral components, including enteric viruses [29], respiratory syncytial virus [30], plant viruses [31], feline calicivirus (FCV-F9), bacteriophage MS2, human papillomavirus (HPV), human immunodeficiency virus (HIV) and different types of coronavirus [32]. Moreover, there are well-established reviews on using chitosan-based bioactive materials in different tissues and organs, such as skin, blood vessels, cornea, cartilage and bone, showing the promising future for fixation, repair and regeneration applications [33, 34]. Finally, Cavaleiro [35] presents a drug delivery system where chitosan in combination with solutions, colloidal systems, microencapsulation systems and coated systems is applied in eye therapy. The study showed the biodegradable, biocompatible, non-toxic polycationic polymer characteristics as well as the antimicrobial and healing properties of chitosan.

Furthermore, the chitosan selected for this study has several reactive functional groups. The fact that it has its polymeric structure already in the pre-processing stage in the manufacture of nanoparticles removes the allergenic potential [36]. The cationic or polycationic form confers bacterial, fungal and viral inhibition properties when interacting with negatively charged residues through adsorption of macromolecules present on their surfaces [37, 38]. The polysaccharide alters cell permeability when chelating metal ions across the cell membrane of microorganisms; as for fungi, the polysaccharide binds its DNA inhibiting RNA synthesis [30, 38]

In addition, chitosan is generally recognized as safe (GRAS) by the US Food and Drug Administration (USA, FDA) and has been used in the distribution of drugs by oral, ocular, pulmonary, nasal, mucous, buccal or vaginal routes, being considered a promising new tool against viral infections. According to a review by Boroumand et al., the therapeutic potential of chitosan nanoparticles against various viral infections was demonstrated [39].

In this scenario, using a translational health research model, our group developed a respirator that is a disruptive innovation that combines the constructive structure of the N95/PFF2 model with a chitosan nanofilm to increase its protective effect [40]. Notably, this technology and its productive processes are entirely national, with all necessary inputs obtained in Brazil as well as all production steps being executed nationally.

Therefore, we assessed the physical–chemical parameters of the chitosan nanoparticles applied to the filtering element of this novel respirator. Furthermore, we evaluated the respirator’s performance in terms of tear resistance, particle filtration efficiency, resistance to breath and flammability, as well as properties related to biological systems interaction (cytotoxicity, antimicrobial and antiviral activity). To the best of our knowledge, this is the first study investigating the association of a semi-facial respirator with chitosan nanoparticles.

## Results and discussion

In our research, the control group consisted of a similar respirator, with the only difference being the absence of chitosan nanofilm impregnation in the filtering element. The novel respirator, which includes the chitosan nanofilm, is referred to as ‘SN95 + CNP,’ while the control respirator is referred to as ‘SN95.’

### Physicochemical characterization

#### Chitosan nanoparticles

Chitosan, a polysaccharide derived from the crustacean exoskeleton, is used in biological applications as a drug carrier or in tissue engineering solutions, because it is degraded at a reasonable rate, without causing inflammatory reactions or producing toxic products [41, 42].

The cationic characteristic of chitosan is considered an attractive factor for addressing types of viruses, fungi and bacteria that have negative surface charges, since it can act as a viral and microbial adsorption and inactivation surface [43].

In this study, chitosan nanoparticles (ChiNPs) were applied as a film on the melt-blown filter element in the intermediary layer of the SN95 + CNP respirator. The physical and chemical characterization of the obtained nanoparticle formulation is described in the present section.

The ChiNPs presented characteristics of small particles (HD of 165.2 ± 35 nm) as described elsewhere [43–46]. The low PDI value, 0.36 ± 0.03, indicates monodispersity of the nanoparticles [47, 48]. Moreover, the ZP showed an average value of 47.5 ± 1.7 mV, so it can be considered that this suspension is stable, with no tendency to flocculate [49]. As expected, the positive charge of ChiNPs is related to dissolution in acetic acid, which provides the protonation of the amino groups [50]. Over a period of 4 weeks at low temperature, the ChiNPs remained stable with respect to HD (109.33 ± 3.74 nm), PDI (0.36 ± 0.06) and ZP (36.57 ± 4.47 mV). Some studies presented similar results [50–53]. These size and surface properties make ChiNPs useful for the treatment of bacterial biofilms and viruses due to their ability to penetrate biofilms and bind easily to the microbial cells within.

**Fig. 1:**
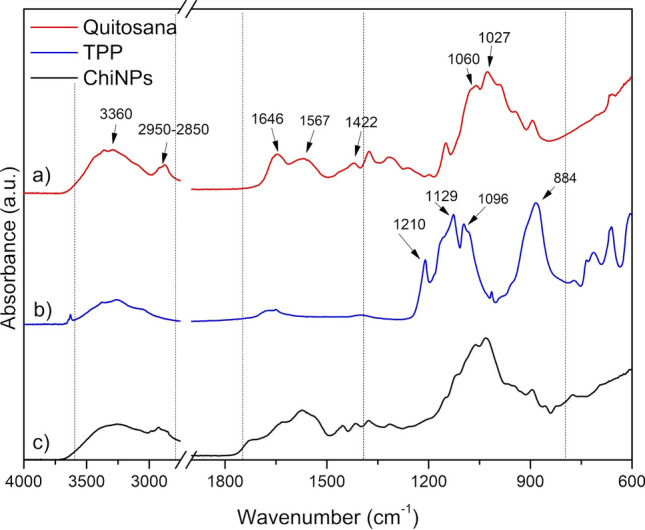
Infrared spectra of **a** chitosan, **b** sodium tripolyphosphate (TPP) and **c** chitosan nanoparticles (ChiNPs). As indicated by the arrows, the 3360 cm^−1^ band represents –OH vibrations and overlapping of the symmetric and asymmetric vibration bands of –N–H of primary amides; 2950–2850 cm^−1^ represents –C–H of aliphatic carbons; 1646 cm^−1^ represents vibration C=O belonging to the carbonyl group (–NHCOCH_3_); 1549 cm^−1^ is related to symmetric angular deformation of the amine group (–NH_2_); 1422 cm^−1^ vibration of the amino group (–N–H); 1060 cm^−1^ attributed to primary alcohol vibration (–C–OH); 1027 cm^−1^ attributed to C–O–C vibration. Vibrations in 1210, 1129, 1096 and 884 cm^−1^ correspond, respectively, to the stretching vibration of the P=O group, symmetrical and asymmetric vibrations of the groups –PO_2_ and –PO_3_ and asymmetric stretching vibration of the P–O–P connection. Vibrations at 3360 and in the range 2950–2850 cm^−1^ correspond to the –OH, –N–H and C–H connections, characteristic of chitosan

To study the chemical characteristics of the obtained ChiNPs, Fourier transform infrared spectroscopy (FTIR) was performed. Figure 1 shows the infrared spectra of chitosan, TPP and ChiNPs.

In Fig. 1a, the 3360 cm^−1^ bands characteristic of the –OH vibrations and overlapping of the symmetric and asymmetric vibration bands of –N–H of primary amides can be observed; 2950–2850 cm^−1^ characteristic of –C–H of aliphatic carbons; 1646 cm^−1^ corresponding to vibration C=O belonging to the carbonyl group (–NHCOCH_3_); 1549 cm^−1^ related to symmetric angular deformation of the amine group (–NH_2_); 1422 cm^−1^ vibration of the amino group (–N–H); 1060 cm^−1^ attributed to primary alcohol vibration, –C–OH and 1027 cm^−1^ attributed to C–O–C vibration [54]. Figure 1b shows vibrations in 1210, 1129, 1096 and 884 cm^−1^ which correspond to the stretching vibration of the P=O group, symmetrical and asymmetric vibrations of the groups –PO_2_ and –PO_3_ and asymmetric stretching vibration of the P–O–P connection. For Fig. 1c, vibrations at 3360 and in the range 2950–2850 cm^−1^ correspond to the –OH, –N–H and C–H connections, characteristic of chitosan. The –OH band becomes wider, indicating a reduction in the number of hydrogen bonds present in ChiNPs due to a cross-linking with TPP [54–57]. Between 1210 and 1096 cm^−1^, there is an overlap of the bands of –C–OH (primary alcohol) and C–O–C stretching, present in the chitosan due to the –PO_2_ and –PO_3_ vibrations from the TPP [44, 58]. An analogous effect can be observed in the vibration at 899 cm^−1^, characteristic of the group P=O, which overlaps with the vibration –C–H present in chitosan. The vibrations located in the 1646–1422 cm^−1^ region correspond to the carbonyl group, the amide (NH_2_CH_3_) and the amino group (NH_2_), characteristic of chitosan. The reduction in the intensity of these vibrations can be attributed to the presence of cross-links between TPP and chitosan [50]. In addition, we noted the reduction of vibrations located in 3400–3200 cm^−1^ of the ChiNPs compared to the chitosan spectrum, suggesting the performance of ionic gelatinization between the protonated amine (–NH^3+^) and the TPP anion [47].

### Mechanical characterization

#### Tensile test

**Fig. 2:**
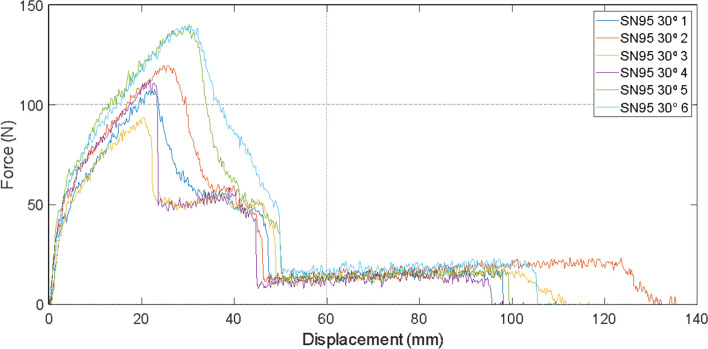
Displacement × force graph of samples taken with a 30° orientation from SN95 respirator

**Fig. 3:**
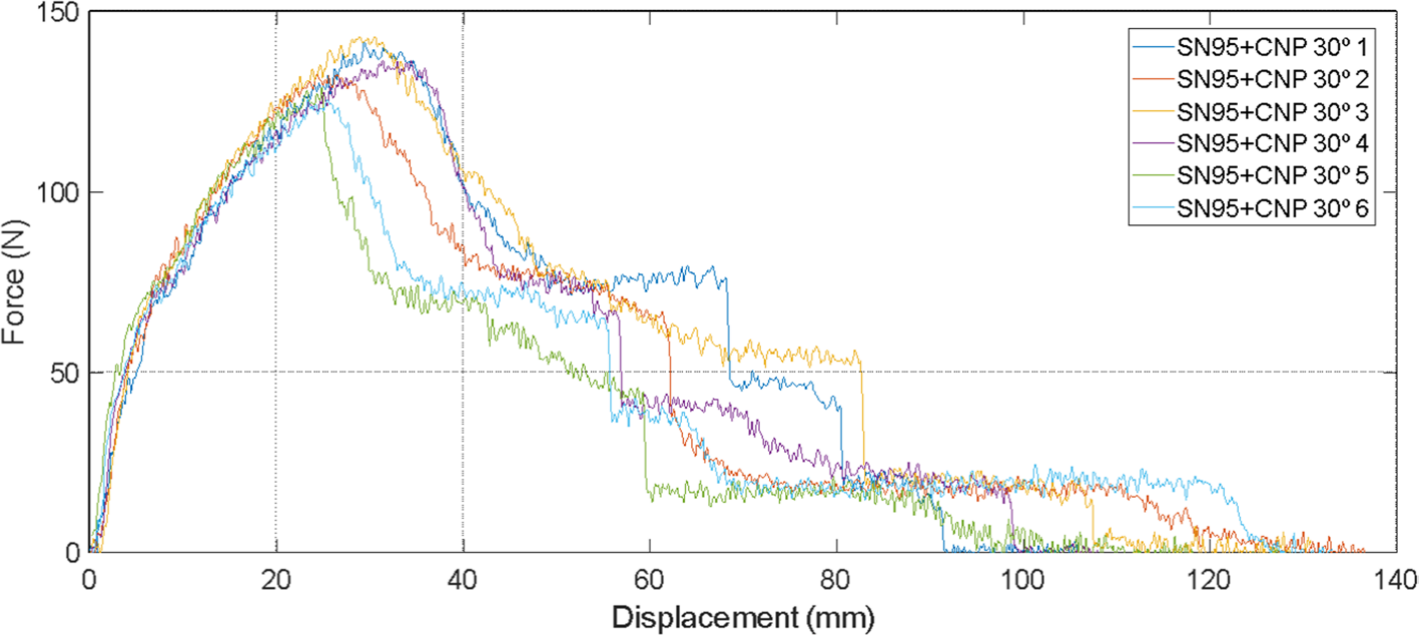
Displacement × force graph of samples taken with a 30° orientation from SN95 + CNP respirator

**Fig. 4:**
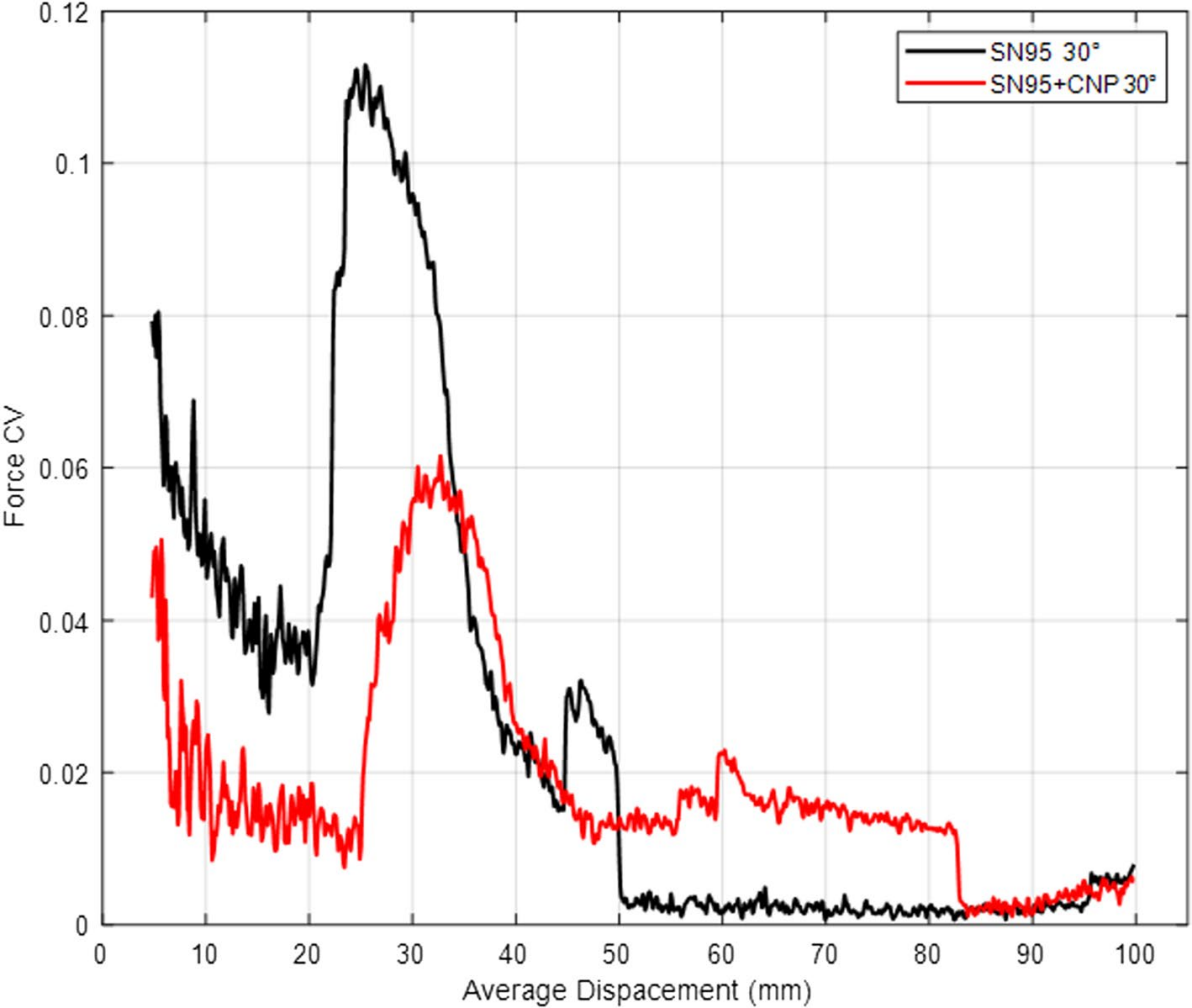
Tensile test coefficient of variation (CV) of samples taken with a 30° orientation from SN95 and SN95 + CNP respirator

To demonstrate compliance of the proposed semi-facial respirator with the industrial standards of PFF2 in vogue in the current market, and the effects linked to the proposed application of nanoparticle in the composition of the respirator, longitudinal tension tests for cut removed with 30° orientation were carried out. Several directions of cuts in the respirator were considered to ensure the analysis of the mechanical properties and verification of the anisotropy of the material [59, 60]. The test consisted of six specimens (samples) for the control model SN95, and another six for the proposed model SN95 + CNP, with 300 mm/min as the traction speed in the test. The results of the longitudinal tension tests of said samples are shown in Figs. 2, 3 and 4, respectively.

It was observed that the average mechanical resistance was 115 N for non-coated SN95 and 135 N for the SN95 + CNP, with the maximum coefficients of variation (CV) for the SN95 and SN95 + CNP around 11% and 6%, respectively, relative to the average. The tests were performed with a longitudinal cut and with a 30° oriented cut, and upon analyzing the graphs, it is possible to verify that the SN95 + CNP is the respirator with higher average mechanical resistance and does not cause material damage in the tension tests applied to the control respirator.

#### Tearing strength

**Fig. 5:**
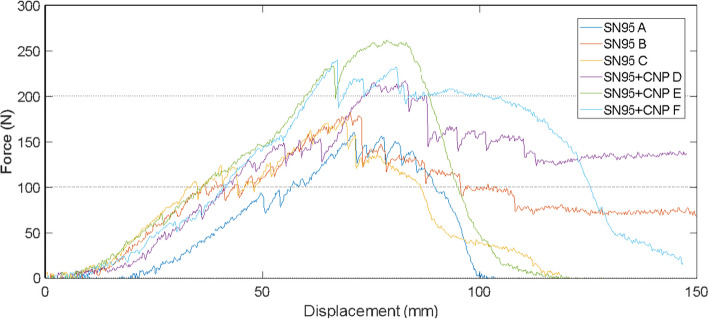
Tearing Strength: SN95 samples (**A**–**C**) and SN95 + CNP samples (**D**–**F**)

**Fig. 6:**
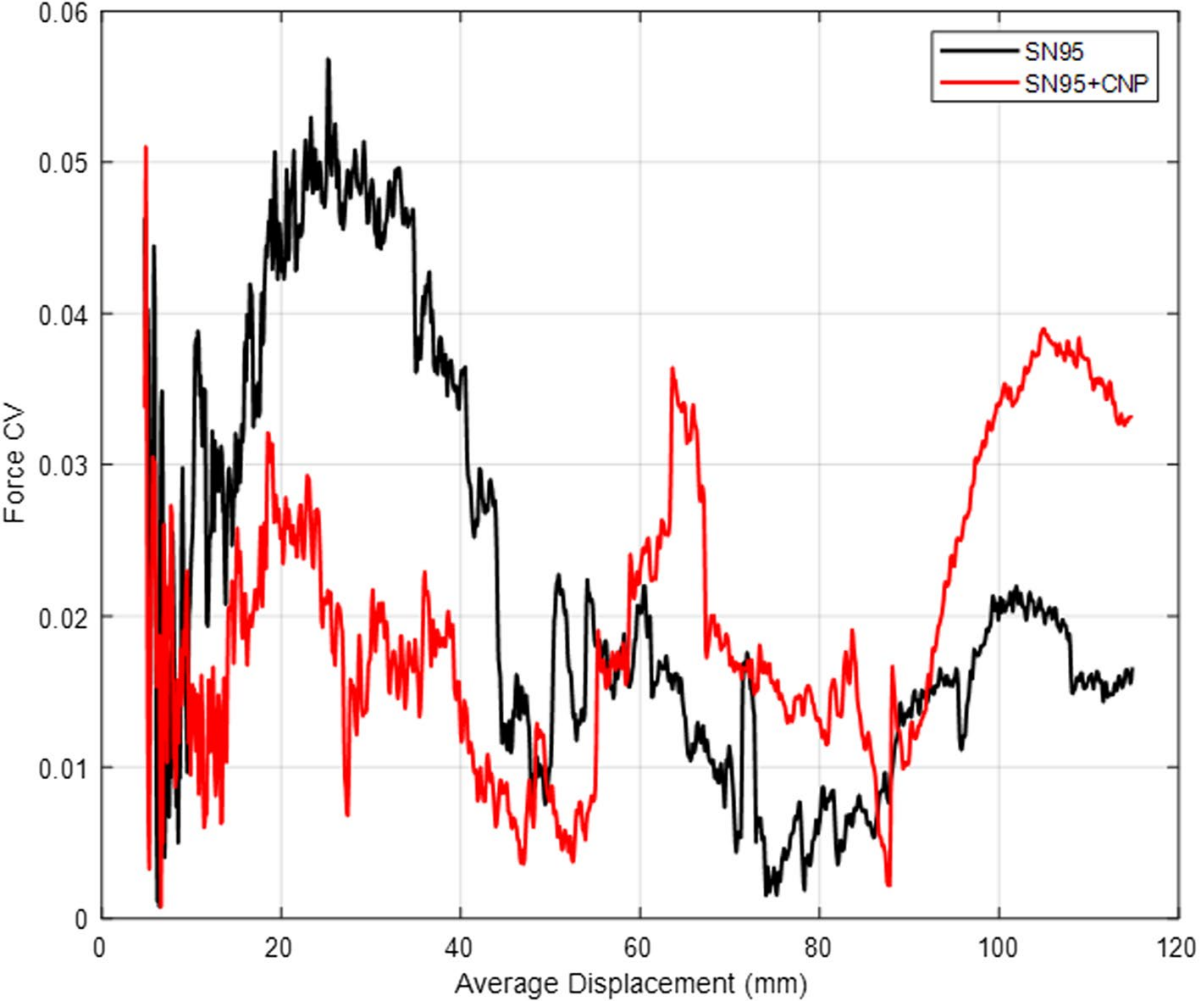
Tearing strength test coefficient of variation of samples taken with a 30° orientation from SN95 and SN95 + CNP respirator

The tearing strength test consists of evaluating the quality of the seams of SN95 + CNP respirators. Six respirators were taken for the experimental procedure, three being SN95 + CNP and three SN95. Figures 5 and 6 present the force x displacement graphs obtained for each respirator and the coefficient of variation of samples (CV), respectively.

A maximum CV of approximately 5% was observed in both SN95 and SN95 + CNP samples. The obtained CV values can be considered reasonably low, indicating the robustness and reliability of the performed mechanical tests.

According to the results, it is possible to conclude that the SN95 + CNP respirator displayed superior seam quality in comparison with SN95. The main reason for this difference can be the presence of additional material immersed with chitosan in the intermediary layer. It is worth mentioning that none of the SN95 + CNP respirators presented damage in the seam region.

#### Filtration efficiency

Measuring the filtration efficiency of a face mask is essential to attest that it meets the minimum standards of particle filtration required for its expected purpose [61].

Table 1 shows the filtration efficiency measurements for the SN95 and SN95 + CNP respirators.

**Table 1:** Mean and standard deviation (in parenthesis) values for filtration efficiency of the respirators without chitosan nanoparticles (SN95) or with chitosan nanoparticles (SN95 + CNP), submitted to: no conditioning, thermal conditioning or vibration conditioning

Respirator	Filtration efficiency (%)		
	No conditioning	Thermal conditioning	Vibration conditioning
SN95	96.64 (0.20)	98.72 (0.29)	96.96 (0.10)
SN95 + CNP	99.10 (0.28)*	96.52 (0.52)*	99.00 (0.11)*

*p < 0.05, calculated by Student’s *T* test

Based on the standards stated on the NBR 13698-2011 by the Brazilian Association of Technical Standards, both respirators met the minimum requirements for filtration efficiency (94%) for use as non-powered air purifying respirator, as well as the international standards NIOSH TEB-APR-STP-0059 (≥ 95% for N95 FFR), EN 149 + A1 (≥ 94% for FFP2) and GB 2626–2006 (≥ 95%) [62].

The SN95 + CNP presents superior filtration efficacy than the SN95, with statistically significant difference (*p* < 0.05), except when previously subjected to thermal treatment (70 °C ± 3 °C for 24 h, room temperature for 4 h, then − 30 °C for 24 h, and return to room temperature for 4 h). However, in practical and realistic contexts of usage, in which the ambient temperature is not as extreme as in the thermal conditioning test, it is expected that the SN95 + CNP respirator should present a filtration efficiency similar to the value obtained without previous conditioning.

#### Resistance to breath

Also known as pressure differential or pressure drop, this parameter indicates the breathability of the mask, calculated by the difference between the air pressure inside and outside the respirator. A low-pressure differential enables air to pass through the filter material with ease, resulting in a more comfortable breathing experience. When considering a specific experimental setup, reducing the air velocity will decrease the pressure differential, while increasing the thickness of the filter material will raise the pressure differential [62].

The average breathing resistance for non-coated SN95 and SN95 + CNP was analyzed and shown in Table 2.

**Table 2:** Mean and standard deviation (in parenthesis) values of breathing resistance, in Pascal (Pa) units, for the respirators without chitosan nanoparticles (SN95) or with chitosan nanoparticles (SN95 + CNP), submitted to: inhalation flows of 30 L/min and 95 L/min, and exhalation flow of 160 L/min

Respirator	Breathing resistance (Pa)		
	Inhalation flow (30 L/min)	Inhalation flow (95 L/min)	Exhalation flow (160 L/min)
SN95	38.33 (6.13)	114.00 (14.70)	139.67 (21.79)
SN95 + CNP	52.67 (1.25)*	136.33 (1.24)	162.00 (2.94)

*p < 0.05, calculated by Student’s *T* test

The maximum values admitted for this test are 70 Pa, 240 Pa and 300 Pa, respectively. Therefore, both SN95 and SN95 + CNP attended the specifications stated in norm NBR 13698-2011, as well as the international norms NIOSH TEB-APR-STP-0059 (max. inhalation flow = 343 Pa; max. exhalation flow = 245 Pa), EU EN 149 + A1 (max. inhalation flow at 95 L/min = 240 Pa) and GB 2626-2006 (max. inhalation flow = 350 Pa; max. exhalation flow = 250 Pa) [62].

There was a statistically significant difference (*p* < 0.05) in the breathing resistance values between SN95 and SN95 + CNP at the inhalation flow of 30 L/min, where the respirator with chitosan nanofilm presented a higher resistance, probably due to an increase in density of the filtering layer caused by the addition of the nanofilm.

#### Flammability

The materials applied in the respirator production must not be highly flammable and must not pose any risk to the user. To assess this parameter, a flammability test is performed. The samples of both categories of respirator, SN95 and SN95 + CNP, showed no burning after the 5-s test, meeting the specifications described in norm NBR 13698-2011.

### Biological characterization

The use of natural macromolecules, such as chitosan, is frequently investigated in efforts against viral outbreaks, including COVID-19, and represents a promising alternative to the design of new diagnostic kits and protective personal equipment, like face masks or respirators [51]. The cationic characteristic of chitosan is considered an attractive factor for addressing types of viruses, fungi and bacteria that have negative surface charges, since it can act as a viral and microbial adsorption and inactivation surface [44].

Before evaluating the antiviral and antimicrobial effect of SN95 + CNP, it was necessary to confirm the biocompatibility of this respirator when exposed to different non-tumoral cells such as keratinocytes (HaCaT) and virus host cells (Vero CCL-81 and Vero E6 cells). Then, antiviral and antimicrobial assays were performed as described as follows.

#### Cytotoxicity assays

**Fig. 7:**
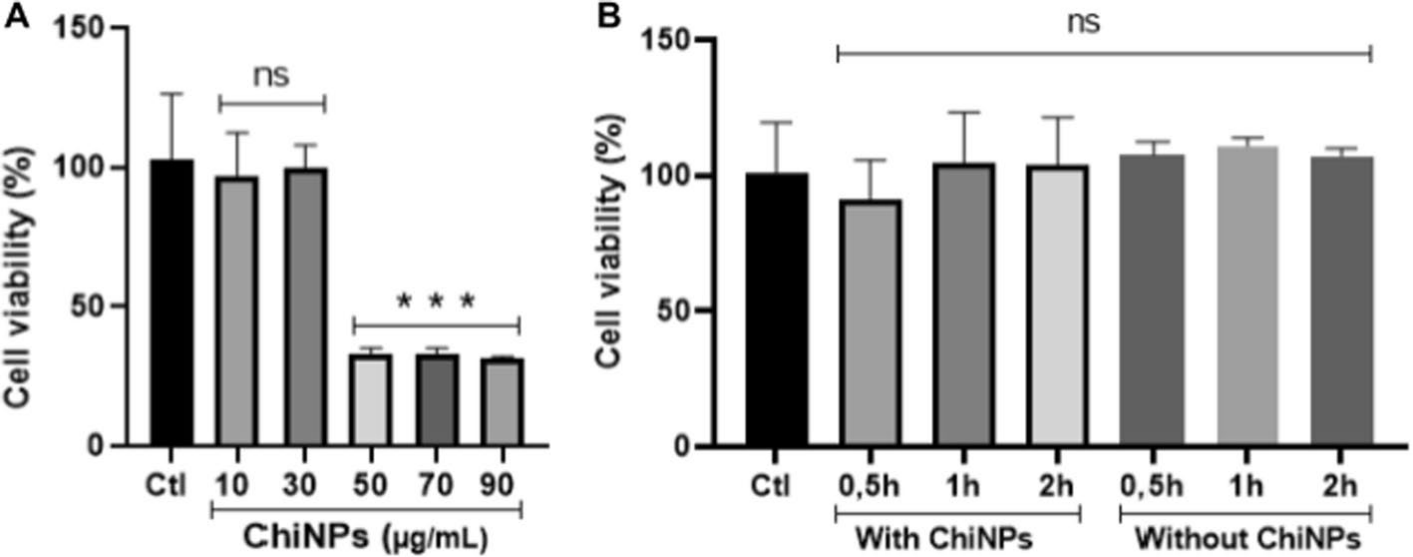
Viability of human keratinocyte cells (HaCaT) after treatment for 24 h with chitosan nanoparticles (ChiNPs) at different concentrations (10, 30, 50, 70 and 90 μg/mL) (**A**), and 24 h with culture medium pre-incubated with fragments of respirators with (SN95 + CNP) and without (SN95) chitosan nanoparticles (0.5, 1 and 2 h) (**B**). ‘Ns’ indicates no statistically significant difference (p < 0.05), while asterisk (*) indicates *p* < 0.05 compared to control and ‘ns’ indicates no significant difference (*p* > 0.05). The results are presented as mean ± SD (*n* = 3)

The cytotoxicity of ChiNPs in suspension and the masks NWF was investigated on HaCaT cells for 24 h, and the results are shown in Fig. 7. Higher concentrations (50, 70 and 90 μg/mL) led to decrease of cell viability (approximately 60%, *p* < 0.001) when compared to the control group. Lower concentrations of ChiNPs (10 and 30 μg/mL) did not induce toxicity (Fig. 7A).

Fragments of non-coated SN95 and SN95 + CNP were incubated in culture media and kept under agitation for 0.5, 1 and 2 h. Chitosan nanoparticles present in the respirator were washed during this process and remained in the culture medium. It is relevant to mention that the concentration of chitosan applied onto the intermediary layer of the respirator was 30 µg/mL.

MTT assay was performed to detect cell viability after incubation of HaCaT cells for 24 h with culture media exposed to fragments of the respirators for different times. After the designated time of exposure, no significant difference was noted between treatment and control (considering as 100% of viability) groups (Fig. 7B). Therefore, it was concluded that SN95 + CNP does not present toxicity to human keratinocyte cells, and meets the criteria of the minimum cell viability value determined by ISO 10993-5: 2009, which is 70%.

The use of natural macromolecules, such as chitosan, is frequently investigated in efforts against viral outbreaks, including COVID-19, and represents a promising alternative to the design of new diagnostic kits and protective personal equipment, like face masks or respirators [63]. Additionally, it has been reported that chitosan nanoparticles were used for wound healing through chitosan nanofiber hydrogel, which, by grafting, favored the repair of sciatic nerve defects in rats with increased nerve regeneration results and promoted vascular penetration [64, 65] and as nanocarriers for drugs or cosmetics [66] due to its biocompatibility, suggesting absence or low toxicity in skin cells.

Hafner et al. [67] reported that nanoparticles based on lecithin and chitosan (proportion of 20:1) were non-cytotoxic for human keratinocyte (HaCaT) and human fibroblasts (BJ) exposed to concentrations under 200 µg/mL for 2 h. Corroborating that, chitosan nanoparticles did not show any significant cytotoxicity against keratinocytes (HaCaT) after 24 h of exposure at 2.5, 5 and 10 µg/mL [68].

**Fig. 8:**
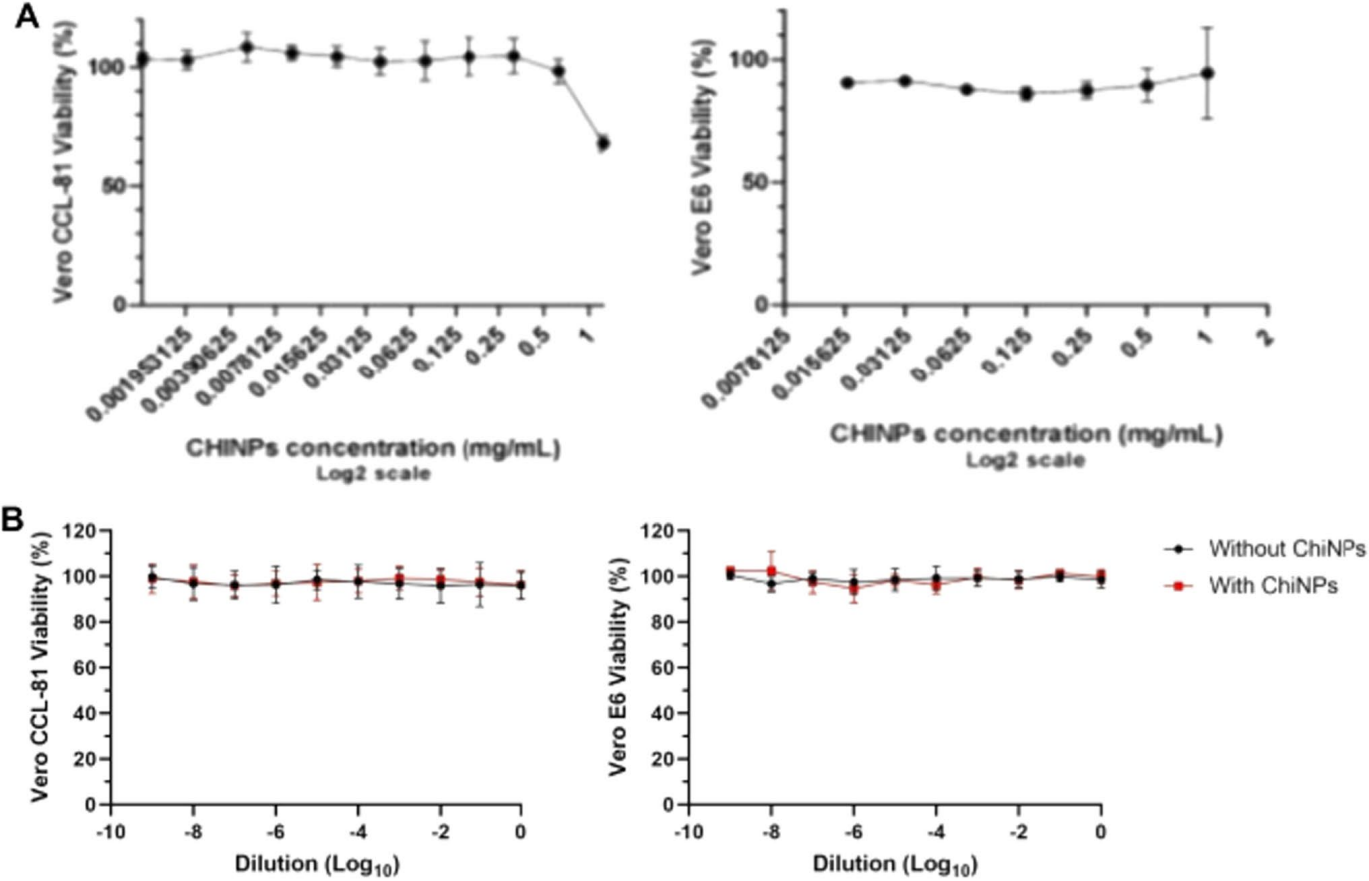
Cell viability of Vero CCL-81 and Vero E6 cells at different concentrations of chitosan nanoparticles (ChiNPs; CCL-81—1.25 to 0.001 mg/mL; E6—1 to 0.016 mg/mL) (**A**), and after treatment for 72 h with culture media pre-incubated with fragments of SN95 (without ChiNPs) and SN95 + CNP (with ChiNPs) respirators (**B**). Pre-incubation conditions: fragments were immersed in 20 mL of culture medium and homogenized by vortex 5 times for 5 s (each) at room temperature. The results are presented as mean ± SD (*n* = 3)

The viability of Vero CCL-81 and Vero E6 cells after exposure to logarithmic dilutions of ChiNPs and of recovered media from the respirators was assessed using resazurin reagent (Fig. 8). The cytotoxicity profiles of ChiNPs (Fig. 8A), and the respirators SN95 and SN95 + CNPs (Fig. 8B) in Vero E6 and Vero CCL-81 cells are shown below. It was observed that concentrations of up to 1 mg/mL of ChiNPs in suspension are not toxic for these cell lines.

Media pre-incubated with SN95 and SN95 + CNP respirators were not toxic to Vero cells (approximately 100% viability) and cytotoxicity was similar between respirators and among dilutions (*p* value > 0.05).

Vero cells are important in vitro models for airway-transmitted viral infections, such as for Influenza virus [69] and SARS-CoV-2 [70]. The non-toxicity of the media exposed to the respirators with and without chitosan nanoparticles assures the possibility of studying the antiviral activity of SN95 + CNP respirator with safety potential.

It is also important to note that chitosan is biocompatible and has been approved as safe (Generally Recognized as Safe—GRAS) by the FDA (Food and Drug Administration, USA) [71]. This status is in line with a systematic review containing a compilation of more than 30 studies [72], on the safety of chitosan as an excipient for medications and dietary supplements, tested in vitro and in vivo by various routes of administration (oral, parenteral, subcutaneous, intramuscular, ocular, intranasal). The review concluded that chitosan is safe for non-parenteral administration and without contact with blood, due to its hemostatic capacity. Accordingly, our experiments tested the cytotoxicity of both respirators with or without chitosan demonstrating that nanoparticles are aligned with the literature. Thus, the present work shows that chitosan nanoparticles, used in the intermediary layer of the SN95 + CNP respirator, potentially present low risk to the future human users’ health.

#### Microbiological evaluation

**Fig. 9:**
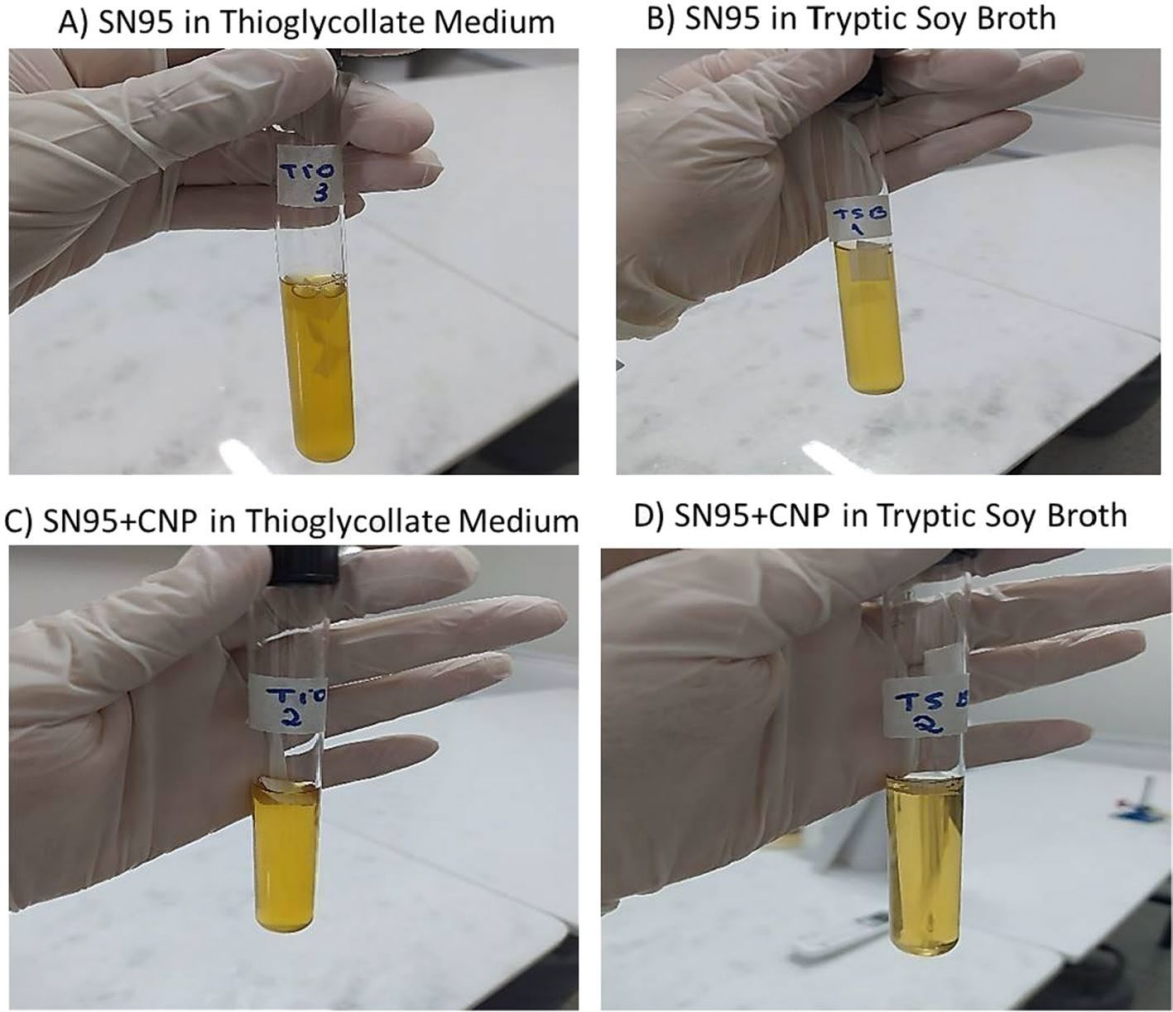
Results of the medium turbidity test of sample of SN95 and sample SN95 + CNP in thioglycolate medium and tryptic soy broth. **A** represents the non-coated SN95 incubated in thioglycolate medium; **B** represents the non-coated SN95 incubated in tryptic soy broth; **C** represents the SN95 + CNP (with ChiNPs) incubated in thioglycolate medium; and **D** represents the SN95 + CNP (with ChiNPs) incubated in tryptic soy broth. Concentration of ChiNPs on the NWF: 30 µg/mL

The antimicrobial effects of SN95 and SN95 + CNP (Fig. 9) were interpreted based on the comparison of turbidity between the samples, according to the McFarland scale [73–75]. The bacteria and fungi that eventually grew in the media were not of a specific strain, but those which were already present on the fabric’s surface and were able to grow with the nutrients and conditions offered by each sample and selective medium.

According to the results in the medium turbidity test, the sample of SN95 presented medium with turbidity of 1.0 on the McFarland scale, for both anaerobic (thioglycolate medium) and aerobic bacteria and fungi (tryptic soy broth) (Fig. 9A, B). On the other hand, the sample of NWF with chitosan nanoparticles and corona treatment (SN95 + CNP) presented a medium with turbidity of 0.5 on the McFarland scale for anaerobic microorganisms (thioglycolate medium), and clear medium (no turbidity) for fungi and aerobic bacteria (tryptic soy broth) (Fig. 9C, D). In summary, the sample of SN95 + CNP was effective to significantly inhibit the growth of microorganisms [74, 75].

Previous studies showed the antimicrobial potential of chitosan. For example, Santos et al. [76] performed an assay with different concentrations of essential oils of clove (CEO) and tea tree (MEO) with chitosan (CS), which were applied to the Gram-positive bacteria *Staphylococcus aureus*, the Gram-negative microorganisms *Escherichia coli* and the yeast *Candida albicans*. In that study, chitosan (0.7 g per 70 mL of 1% acetic acid solution) induced the inhibition of bacterial and fungal growth (inhibition halos between 7.0 and 8.5 mm) of the three strains in the disk diffusion assay, confirming its antimicrobial property. Another study showed that chitosan can be applied as antimicrobial films in food packaging [77].

Sun et al. [78] studied the antimicrobial activity of positively charged electret filters made of nylon blended or impregnated with chitosan against *E. coli* and *Bacillus subtilis*. They showed that the filters presented antimicrobial activity*.* They also demonstrated that when chitosan is not blended, but added to the surface of the filter material, it presents greater antimicrobial activity due to more NH_3_^+^ groups availability being free to interact with the negatively charged surface of bacteria, causing, thus, the membrane disruption and cell lysis. This study could explain why our SN95 + CNP respirator presented antimicrobial activity since the intermediary layer has chitosan nanoparticles and the NWF was electrified with corona treatment.

Additionally, Dyvia et al. [38] tested the antimicrobial and antibiofilm activity of chitosan nanoparticles against *Klebsiella pneumoniae*, *Escherichia coli*, *Staphylococcus aureus*, *Pseudomonas aeruginosa* and found a minimum inhibitory concentration (MIC) of 100 mg/mL for *K. pneumoniae*, *E. coli* and *S. aureus* and 200 mg/mL for *P. aeruginosa*. In addition, the electrostatic interaction with the cell membrane and lysis with extravasation of bacterial cytoplasm were identified as possible mechanisms of action. In that same study, the authors confirmed that chitosan also had antibiofilm activity against the studied strains. However, the concentration of nanoparticles applied in SN95 + CNP in this assay (30 µg/mL) impaired bacterial and fungal growth. Recently, Abdel-Razek [79] reported that the MIC of chitosan nanoparticles against the studied bacteria was between 0.156 and 2.5 μg/mL and the largest inhibition zone was obtained at a concentration of 20 μg/mL. *Aeromonas sobria*, *A. hydrophila* and *Pseudomonas aeruginosa* were the most susceptible bacterial strain, while *Staphylococcus aureus* and *Pseudomonas fluorescens* were the most resistant.

#### Evaluation of the antiviral activity

A significant reduction of 2.0 logarithms was observed in the viral titration, which corresponds to the determination of antiviral activity related to a 99.00% reduction in viral particles when betacoronavirus (MHV-3) and influenza (H1N1) were incubated with SN95 + CNP. Regarding the alphacoronavirus virus (CCoV), a reduction of 1.5 logarithms (Mv = 1.5) was observed, which corresponds to a reduction of 96.83% of viral particles after 120 min of contact with SN95 + CNP. Thus, the novel respirator (SN95 + CNP) reduced 99% of H1N1 virus and MHV-3 betacoronavirus particles and 96.83% of CCoV alphacoronavirus compared to the conventional respirator (SN95) (Table3).

**Table 3:** Results of viral titration of MHV-3 (betacoronavirus), CCoV (alphacoronavirus) and H1N1 (Influenza), after 120 min of contact with SN95 + CNP, evaluated with the results of antiviral activity (Mv) and the relation in percentage of viral reduction. Concentration of ChiNPs on the NWF: 2.5 mg/mL

	Methodology applied	Control group viral titration	SN95	SN95 + CNP	Standard deviation	Reduction in titration (log)	Reduction in percentage (%)
CCoV	Cytopathic effect	10^5^	10^4.5^	10^3^	10^0.5^	1.5	96.83
MHV-3	Cytopathic effect	10^5^	10^5^	10^3^	10^0.5^	2	99.00
H1N1	RT-qPCR (Cq)	27.1	27.5	29.5	0.5	2	99.00

Therefore, according to the results obtained, it is shown that the SN95 + CNP respirator (ChiNPs concentration = 2.5 mg/mL) has antiviral activity against the Influenza (H1N1) and alphacoronavirus and betacoronavirus.

Based on the results, the use of chitosan as a filter element for semi-facial respirators has the potential to increase the effectiveness of this type of PPE. This aspect is relevant, especially in terms of preventing contamination of health workers by various microorganisms, including coronaviruses.

In previous studies, it was demonstrated that silver nanoparticle/chitosan composites presented activity against the influenza virus of H1N1, especially with nanoparticles smaller than 10 nm average diameters [80]. Another study showed that the conjugation of an influenza A (H1N1) antigen to the surface of N-trimethylaminoethylmethacrylate chitosan (TMC) nanoparticle induced immune responses against the virus [81].

Furthermore, a study carried out specifically for viruses demonstrated that chitosan chloride *N*-2-hydroxypropyl-3-trimethylammonium (HTCC), with different degrees of substitution, acts as effective inhibitors of all low pathogenic human coronaviruses (HCoV-NL63, HCoV-OC43, HCoV-229E and HCoV-HKU1), which circulate around the world and generally cause the common cold. The cationic charge of chitosan favors electrostatic interaction with the surface of viruses, which have a negative surface charge [82, 83]. These data indicate that chitosan and its cationic derivatives have an inhibitory effect against a broad range of coronavirus’ strains. The inhibition mechanism is related to the interaction between chitosan and the coronaviruses’ surface protein and electrical surface charge [53, 84]. Similar results that reiterate the antiviral activity of chitosan were also described by Lan et al. [85]. It was shown that the antiviral and antibacterial activity of chitosan oligosaccharide modified cellulosic fibers, which presented a reduction of the viral load by 99.19% after 1 h of contact of the bacteriophage MS2—used a surrogate of the SARS-CoV-2 and other pathogenic viruses—and a bacteriostatic rate of 94.4% against *Staphylococcus aureus* (ATCC6538) and 85.9% against *Escherichia coli* (ATCC8739).

Therefore, results presented in this work corroborate the hypothesis that chitosan-based nanomaterials present antimicrobial and antiviral activity and it is safe for use in medical devices and PPEs, agreeing with preexistent studies in the literature.

The production of chitosan nanoparticles and incorporation onto the filtering element (intermediary layer) of the novel semi-facial respirator (SN95 + CNP) was successful, resulting in a PPE produced within all the normative parameters necessary for approval by regulatory agencies (such as Brazilian ANVISA) for commercialization.

Additional benefits of the chitosan application include collaborating with Brazilian national industry of PPE and numerous devices, aligned with the concept of *circular economy* [86] by making the best use of wastes generated by crustacean consumption.

## Conclusion

The novel semi-facial respirator containing a monodisperse system with highly positive electrically charged chitosan nanoparticles was superior to the commercially available respirator in terms of tearing strength. Both the SN95 and SN95 + CNP respirators met the requisites of aerosol penetration, resistance to breath and flammability, necessary to be considered a safe PPF2 for health care use, according to the norm adopted by the Brazilian regulation agency—ANVISA. The novel respirator did not present toxicity to keratinocytes (HaCaT) nor to monkey kidney cells (VERO CCL-81 and E6) but presented significant antibacterial and antiviral capacity. Our findings suggest that the novel SN95 + CNP respirator is a promising alternative in terms of quality and protection, due to its innovative technology. The upcoming results of our ongoing clinical research may soon provide supporting evidence of the SN95 + CNP respirator’s potential to inactivate the new coronavirus (SARS-CoV-2). Nonetheless, the limitations of our work could be assessment of the allergic potential of our respirators and of the chitosan nanoparticles, as well as their potential toxicity to respiratory cells. The ChiNPs used in our masks are made from medical grade chitosan, which means they do not contain a protein that induces allergies. Still, it is unlikely that the chitosan nanoparticles in the filtering layer of our SN95 + CNP mask would be able to be inhaled and reach inside the human airways, since the size of the chitosan nanoparticles would be large enough to prevent them to pass through the adjacent NWF layers which may function as barriers between the filtering layer and the user’s face. Therefore, our novel N95 respirator presents improved mechanical, antimicrobial and antiviral properties and can be considered safe.

## Methods

**Fig. 10:**
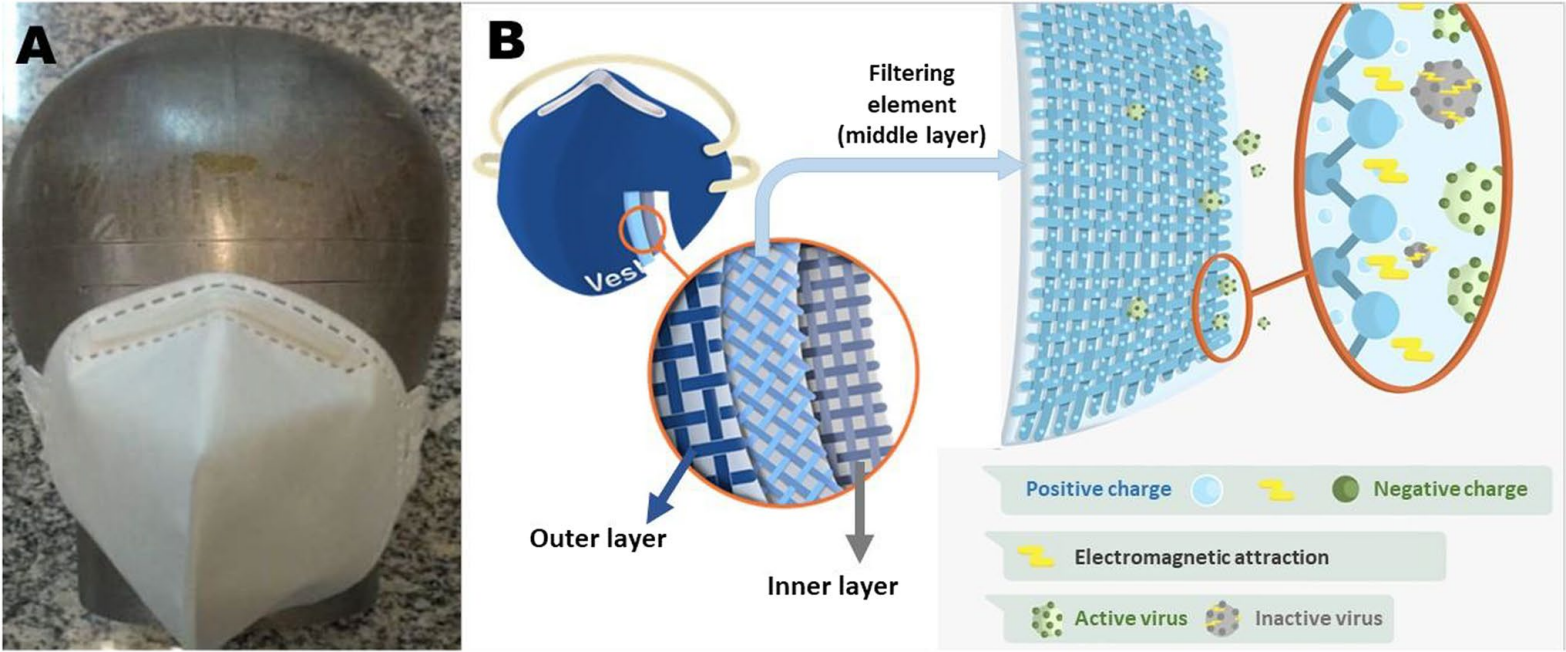
Respirator’s general composition and structure: **A** Photograph showing the frontal view of the respirator on a head mannequin; **B** Schematization of the respirator’s inner, middle (filtering element) and outer layers, and virus inactivation mechanism of the filtering element. *Sources*: **A** company Falcão Bauer, **B** the authors

The object of our research is a novel facepiece respirator, illustrated in Fig. 10. As shown in Figs. 10A, it is characterized by presenting a fold shape and a head strap like the widely used PFF2- or N95-type respirators. The respirator is composed of alternating layers (Fig. 10B) of nonwoven fabric (NWF) and felt, that cover the mouth and nose. In the intermediary layer lies the filtering element, which consists of a NWF impregnated with the chitosan nanofilm. The control group used in our research was a similar respirator, differing only in the fact that the filtering element is not impregnated with chitosan nanofilm. The novel respirator with chitosan nanofilm is hereafter named ‘*SN95* + *CNP*,’ while the control respirator is hereafter named ‘*SN95*.’

### Materials

Medical Grade Chitosan with a medium molecular mass of 198 kDa, deacetylation degree of 76% and insoluble content of < 0.5% was provided by CERTBIO. Tripolyphosphate (TPP) and acetic acid were acquired from Sigma Aldrich and Vetec, respectively; ultrapure water (resistivity of 18.2 MΩ cm) was obtained from a Master System All MS2000—Gehaka System; and nonwoven fabric (NWF) was donated by the MCI Indústria e Comércio de Produtos Eletrônicos Ltda.

### Synthesis of chitosan nanoparticles

The chitosan synthesis method used in this study was the Ionic Gelation Method, reported previously by Antoniou et al. [44] and Tsai [87]. In summary, 0.5 g of chitosan was dissolved in 0.75% (v/v) aqueous acetic acid solution by magnetic stirring (300 rpm) at room temperature (25 °C) for 1 h. The chitosan solution (0.5 mg/mL) was then fragmented using a disperser at 25,000 rpm for 3 h. Subsequently, a 0.7 mg/mL aqueous solution of TPP was added dropwise to the chitosan solution (proportion of 8:1 Chitosan:TPP) under a vigorous disperser stirring (10,000 rpm) and at room temperature (25 °C), using an infusion pump set with an infusion rate of 0.5 mL per minute for 1 h and 15 min. After TPP addition, the solution containing the formed chitosan nanoparticles was left under stirring for 10 min at 10,000 rpm then at 300 rpm for 10 min, at room temperature on both occasions. Larger particles were removed by centrifugation under 10,000×*g* for 30 min at 4 °C. The supernatant containing the chitosan nanoparticles (ChiNPs) was collected and nanoparticle mean diameter was determined by Dynamic Light Scattering (DLS). Finally, the samples were stored under refrigeration (4 °C) and protected from light.

### Characterization of ChiNPs

The hydrodynamic diameter (HD) and polydispersity index (PDI) were determined by DLS, and the *ζ*-potential (ZP) by electrophoretic measurement using an electrode AQ-1214 (Brookhaven's ZetaPals). The analyses were conducted at room temperature (25 °C) in a BI-SCP cell with laser He–Ne operating at 632.8 nm, scattering angle of 90°, average viscosity of 0.88872 mPa s and average refractive index of 1.330. For stability studies, a ChiNPs sample was stored at 4 °C, protected from light and characterized as described for 4 weeks following synthesis. The data were presented as the average ± standard deviation of triplicate measurements.

Fourier transform infrared (FTIR) spectra of chitosan, TPP and ChiNPs were determined using a Spectrum 400 Perkin Elmer. The spectra were obtained in the range of 4000–600 cm^−1^, using 32 scans at a resolution of 4 cm^−1^. A total attenuated reflectance accessory coupled to infrared (IR) spectrum was used. Powder of ChiNPs was obtained by freeze drying. The ChiNPs solution was transferred into Petri dishes (90 cm × 15 cm), frozen in an ultrafreezer (-86 ºC) for 24 h and lyophilized for 72 h.

### Surface modification of the nonwoven fabric with corona discharge and ChiNPs

To facilitate the application of chitosan nanoparticles on the surface of the nonwoven fabric (NWF), corona discharge was applied using the Carnevalli extrusion line. Subsequently, the nanoparticles were introduced using the dip coating method, where the corona-treated NWF was immersed in a solution of nanoparticles of concentration: 30 μg/mL in the first batch, or 2.5 mg/mL in the second batch, and dried. Shortly after drying, the solution of nanoparticles was reapplied by spray on the NWF’s surface using the Air Plus spray (Schutz) and dried with a Fox Ion 3 hair dryer (Taiff).

All the assays, except the evaluation of antiviral activity of the respirators, were performed with the first batch of respirators.

### Mechanical characterization

Mechanical tests were carried out to validate the SN95 and SN95 + CNP technical conformity, according to the requirements of the competent regulation agency. The following are the normative bases:

- ASTM D5035—Standard Test Method for Breaking Force and Elongation of Textile Fabrics (Strip Method).
- ASTM D3776/D3776M—Standard Test Methods for Mass per Unit Area (Weight) of Fabric.
- Brazilian standard ABNT NBR 13.697—Respiratory protective equipment (Particulate filters).
- Brazilian standard ABNT NBR 13.698—Respiratory protective equipment (Filtering semi-facial part for particles).
- Resolution RDC 356 of the Brazilian National Health Surveillance Agency (ANVISA)—Requirements for the manufacture, import and purchase of medical devices identified as priorities for use in health services, due to the international public health emergency related to SARS-CoV-2.

Four property types were tested by the Experimental and Computational Mechanics Group at the University of Brasília—GMEC/UnB—force–deformation ratio, grammage, structural morphology and thermal insulation—while the company *Grupo Falcão-Bauer* tested the particle filtration efficiency, resistance to breath and flammability.

#### Tensile test

It was tested the stretching resistance of the respirator developed by our group that was applied a layer with chitosan nanoparticles (SN95 + CNP), and as control group a similar respirator, differing only in the fact that the filtering element is not impregnated with chitosan nanofilm (SN95). Applying the cut strip test 1C (for NWF) of the equivalent standard mentioned above, six rectangular samples (strips) from each respirator were taken, through a uniform longitudinal cut (30° cut). Samples must be 25 ± 1 mm wide (with at least 20 wires in width) at least 150 mm long. In addition, the long dimension must be precisely parallel to the direction of testing and application of the force.

To perform the strip test, i.e., to determine the force needed to break a specific width of fabric, the force–deformation ratio (N × mm) was evaluated using the equipment Universal Instron 8801, with a 200 N load cell and sensitivity of 2.0028 mV/V. The speed of the tensile test was adjusted to 300 mm/min, generating the point plot of these variables, as well as the average value in the six samples. This is important to indicate the proper resistance of the SN95 + CNP for adaptation to regulatory agencies requirements and factory production.

#### Filtration efficiency

This experiment was conducted according to the standards stated on the NBR 13698-2011 by the Brazilian Association of Technical Standards, which specifies filtering half mask requirements for use as non-powered air purifying respirators.

A flow of 95 L/min of sodium chloride solution passed through the PFF and the concentration of the aerosol was measured before and after it. Aerosol penetration was monitored and recorded at sampling intervals not exceeding 5 min, until the PFF had been exposed to 150 mg of aerosol.

The concentration of this aerosol was measured before and after the PFF under test, by flame photometry or by light scattering photometry. The equipment allowed accurate penetration determinations in the range of 0.001–100%.

For statistical comparison between SN95 and SN95 + CNP, Student’s *T* test was performed by calculating the *F* values followed by *p* values, indicating the significance of statistical difference between the two groups.

#### Resistance to breath

This experiment was conducted according to the standards stated on the NBR 13698-2011 by the Brazilian Association of Technical Standards, which specifies the filtering half mask requirements for use as non-powered air purifying respirators.

The simulator machine was adjusted to 25 cycles/min and 2 L/pump. The PFF was securely adjusted on the dummy head, without causing deformation. The exhalation resistance was measured at the mouth opening of the mannequin, using an adapter. The flow rate at which resistance is measured was corrected to 23 °C and 100 kPa absolute (1 bar). Exhalation resistance was measured by placing the head successively in five positions: looking straight ahead, looking vertically upwards, looking down vertically, leaning to the left side and leaning to the right side.

For statistical comparison between SN95 and SN95 + CNP, Student’s *T* test was performed by calculating the *F* values followed by *p* values, indicating the significance of statistical difference between the two groups.

#### Flammability

This experiment was conducted according to the standards stated on the NBR 13698-2011 by the Brazilian Association of Technical Standards, which specifies the filtering half mask requirements for use as non-powered air purifying respirators.

The PPF was placed in a movable dummy head capable of describing horizontal circles with adjustable speed. The distance between the top of the burner and the lowest part of the PPF that passes through the flame was adjusted to (20 ± 2) mm. With the dummy head outside the area adjacent to the burner, the gas was allowed to flow through and adjusted, through the regulator, with pressure between 20 and 30 kPa. The burner was ignited, and the flame height adjusted to (40 ± 4) mm by action of the existing valve. The flame temperature, 20 mm above the top of the burner, was (800 ± 50) °C. To obtain the correct temperature, it was necessary to adjust the burner air. All equipment was protected from external drafts.

The PPF mounted on the metal head was passed through the flame once, at a speed of (60 ± 5) mm/s, and the effect of the flame on the PPF and its components was recorded.

### Cytotoxicity assay on human keratinocytes

Since the skin is the first barrier of the human body in direct contact with the respirator, and indirectly with the chitosan nanofilm, it was assessed whether our mask NWF and the chitosan nanoparticles are toxic for the human skin cells (i.e., keratinocytes). Therefore, human keratinocytes (HaCaT) were cultured in DMEM—Dulbecco’s Modified Eagle’s Medium—containing 10% of fetal bovine serum and 1% of gentamicin and incubated at 37 °C in a humid atmosphere with 5% CO_2_.

For viability assay, HaCat cells were seeded into 96-well culture plates at a density of 5 × 10^3^ cells/well in DMEM culture medium overnight at incubators’ conditions. Then, cells were incubated with chitosan nanoparticles (ChiNPs) and culture medium exposed to fragments of SN95 and SN95 + CNP, as described below.

Essentially, ChiNPs were filtered through a 0.45 µm membrane and exposed to the cells for 30 min, 1 h, 2 h and 24 h at the concentration 30 µg/mL. The pH of the medium was adjusted to 7. In parallel, fragments (2 cm × 2 cm) of SN95 and SN95 + CNP previously exposed to UV light for 15 min on each side were immersed in culture medium and kept under constant agitation at room temperature for 30 min, 1 and 2 h. Then, the culture medium was filtered (0.22 µm) and exposed to the cells for 24 h. The cells were incubated at 37 °C in a humid atmosphere with 5% CO_2_.

Cell viability assay was performed using a MTT (3- [4, 5-dimethylthiazol-2-yl]-2,5-diphenyltetrazolium bromide) assay. After incubation, the MTT solution (0.5 mg/mL) was added to each well and the cells were incubated at incubators' conditions for 2 h. Then, 150 mL of DMSO was added and the absorbance of the solution present in each well was measured at a wavelength of 595 nm, using a UV–Vis spectrophotometer (Molecular Devices, USA). The control group was considered as 100% cell viability. All tests were performed as triplicates.

ANOVA statistical tests with Tukey’s posttest were used for analysis and graph construction in GraphPad Prism 9 software. To determine statistical significance, a value of *p* ≤ 0.05 was adopted.

### Cytotoxicity assay on Vero cells

Tests of NWF and chitosan nanoparticles cytotoxicity were carried out in Vero CCL-81 and Vero E6 cells because they are the cells used in the model test of antiviral activity used in this study. In general, these assays are carried out to confirm that the product being tested is not cytotoxic for the cells that will be later used in the antiviral assay, thus avoiding distorted results in the analysis. Vero CCL-81 cells were cultured in DMEM supplemented with 10% fetal bovine serum, 10 U/mL penicillin and 10 µg/mL streptomycin at 37 °C in a humid atmosphere containing 5% CO_2_. Vero E6 cells were cultured in Minimum Essential Media (MEM) supplemented with 1 mM sodium pyruvate, 10% fetal bovine serum, 10 U/mL penicillin, 10 µg/mL streptomycin and 250 ng/mL Amphotericin B at incubators' conditions (37 °C in a humid atmosphere with 5% CO_2_).

Vero cells were seeded on 96-well plates at a density of 1 × 10^4^ and 2 × 10^4^ cells/well for CCL-81 and E6, respectively, and incubated at incubators' conditions for 24 h. SN95 and SN95 + CNP respirators were cut into fragments (2 cm × 2 cm) and exposed to UV light for 15 min on each side. Then, fragments were immersed in 20 mL of culture medium and homogenized by vortex 5 times for 5 s (each) at room temperature.

The recovered media from the respirators was serially diluted by a factor of ten (10^−1^ to 10^−9^) and 150 µL of each logarithmic dilution was applied to the wells in quadruplicate. Cells were incubated for 72 h, and cell viability was measured using resazurin (alamarBlue™ Cell Viability Reagent, Invitrogen), according to the manufacturer’s protocol. Cells incubated with culture medium only were used as viability positive control. Absorbance was measured at a wavelength of 570 nm and 600 nm, using the Benchmark Plus microplate spectrophotometer (BIO-RAD). Three independent experiments were performed for each cell line. Two-way ANOVA with Sídák's multiple comparison test was used to compare the cell viability estimates obtained after exposure to the recovered media of each respirator in GraphPad Prism 9 software.

### Microbiological evaluation

For the evaluation of the presence of fungi and bacteria, a microbiological evaluation study was carried out on samples of nonwoven fabric (NWF) of imported origin without corona treatment (TI-WOC) and those that received the corona treatment and subsequent covering with ChiNPs (TI-WIC). The reason for the study to focus only on these samples is that there were just enough materials for the test and to develop the SN95 + CNP respirators. The NWF samples were sectioned in fragments with dimensions of 1 cm × 1 cm, following the specific methodology of the sterility test for health products [73, 74]. The samples were handled in a sterile environment using the Quimis biological safety cabinet, model Q216F21RA1. For the test, Tryptic Soy Broth (TSB—Kasvi, Italy) culture media was used to assess the presence of fungi and aerobic bacteria, and thioglycolate medium (Kasvi, Italy) to assess the presence of anaerobic microorganism, on the respirator’s layers. The results were interpreted based on the comparison of turbidity between the samples, using the McFarland scale.

### Evaluation of antiviral activity for influenza (H1N1) and coronaviruses (MHV-3 and CCoV)

#### Cell culture

In this study, L929 cells (connective tissue fibroblasts) were maintained in culture with DMEM (Dulbecco's Modified Eagle's Medium) with the addition of supplements at 37 °C and 5% CO_2_ and distributed in plates suitable for monolayer culture.

#### Virus cultivation

Three types of viruses were used: betacoronavirus (MHV-3), influenza (H1N1) and alphacoronavirus (CCoV). MHV-3 and CCoV viruses were selected because they belong to the same family as SARS-CoV-2. H1N1 virus was selected because it is a respiratory virus that causes influenza. Concentrations of 10^5^ virus particles, previously calculated, were used. It was determined of the dose for use by the TCID50, according to bellow. The calculation of the *p* value was based on the number of replicates of the titration according to the 7 dilutions in base 10.

$$\begin{aligned} Y & = X \times 10a\quad a = \sum p - 0.5 \times {\text{Dilution base}} - {\text{Base 10 was used}} \\ a & = \sum 6 - 1.0;\;a = 5.0 \\ \end{aligned}$$

#### Sample preparation

The samples of conventional type N95 respirator (SN95) and type N95 respirator with chitosan nanoparticles (SN95 + CNP) were weighed (0.2 g) and cut in 2 cm × 2 cm of each cloth, according recommended in ISO 18184:2019. Then, the cloth fragments were soaked in the solution with the viral particles and the samples were homogenized using vortexing for 5 s every 20 min for 2 h to ensure virus contact with fabric.

#### Cytotoxicity analysis

The cell control group and the SN95 and SN95 + CNP groups were incubated in supplemented culture medium under the same conditions as the virus assay. Culture medium was added to the cell monolayer in duplicate for each group and at a 1:10 dilution. At the end of 24 h, the cell culture of all groups was evaluated by means of images.

#### Analysis of antiviral activity

After counting the exposure times (direct contact), an aliquot was separated into new tubes, ending the contact with the sample. The viral control group was performed in the same way without having contact with any fabric fragment. Afterward, it was inoculated in a monolayer of cells in quadruplicate for each group to evaluate the viral multiplication.

#### Analysis of results

The inoculated cell culture was evaluated according to morphology changes, which are characterized by the cytopathogenic effect caused by the viruses.

The cell control group (no virus) was compared with the viral control group and SN95 and SN95 + CNP groups to assess the presence of viral replication through image capture and comparison with viral titration performed, which demonstrates the reduction in the number of infectious viral particles in logarithms. The result was expressed as a reduction in logarithms of the number of viral particles and transformed into reference values as recommended in ISO 18184:2019.

## Data Availability

The datasets used and/or analyzed during the current study are available from the corresponding author on reasonable request.
